# Case Report: Simultaneous Ascending Aortic Dissection and Pulmonary Artery Dissection Combined by Aortopulmonary Fistula After Aortic Valve Replacement

**DOI:** 10.3389/fcvm.2021.779993

**Published:** 2021-11-18

**Authors:** Lihui Song, Sizheng Xiong, Jun Li

**Affiliations:** Department of Cardiothoracic and Vascular Surgery, Tongji Medical College, Tongji Hospital, Huazhong University of Science and Technology, Wuhan, China

**Keywords:** aortic dissection, pulmonary artery dissection, aortopulmonary fistula, cardiac reoperation, surgical repair

## Abstract

Aortopulmonary fistula with/without pulmonary artery dissection is an extremely rare and fatal complication of acute aortic dissection and is often discovered postmortem. We present a case with a simultaneous ascending aortic dissection and pulmonary artery dissection combined by aortopulmonary fistula after aortic valve surgery. However, the patient died of postoperative complications after surgery. Herein, the anatomical basis for this rare entity and its outcome is explored with an emphasis.

## Introduction

Acute aortic dissection (AAD) is rare with an incidence of 2.6–3.5 cases per 100,000 person-years (1) and so is pulmonary artery dissection (PAD) with only 150 cases reported from 1842 to 2018 (2). When a patient develops both two entities, it is extremely a rare and lethal condition, in particular, ascending aorta involvement [Stanford type A aortic dissection (TAAD)]. There is often some anatomical connection between the simultaneous appearance of TAAD and PAD including patent ductus arteriosus, aortopulmonary fistula, and aortopulmonary window (3–5). In addition to these characteristics, this case was secondary to aortic valve replacement, which was almost not reported in the previous studies.

## Case Presentation

**Figure 1:**
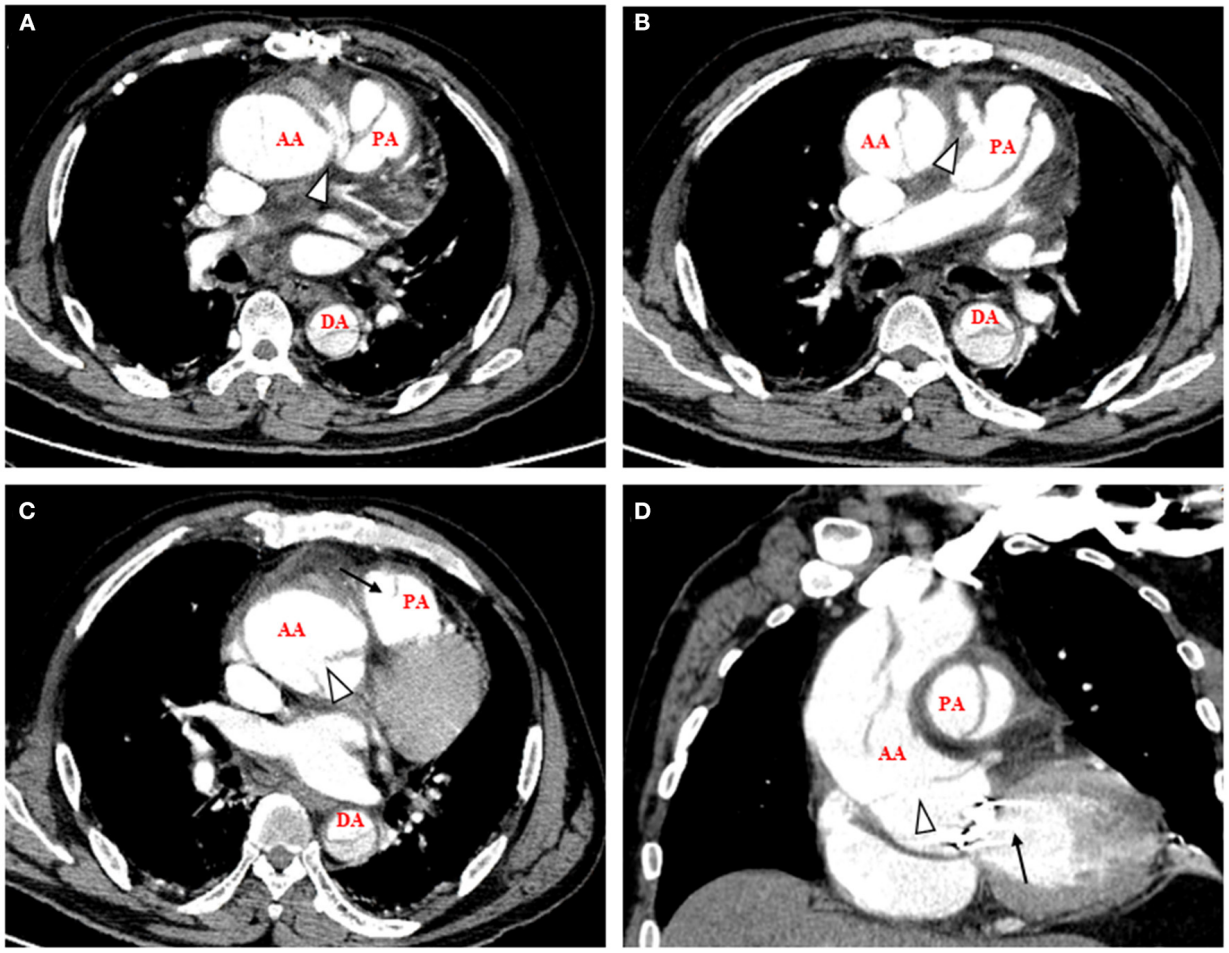
CT angiography on admission showed a simultaneous ascending aortic dissection and pulmonary artery dissection combined by aortopulmonary fistula. **(A)** Axial view, arrowhead showed the aortopulmonary fistula near the aorta root. **(B)** Axial view, arrowhead showed the aortopulmonary fistula near the PA. **(C)** Axial view, arrowhead showed the intimal flap of AA and arrow showed the intimal flap of PA. **(D)** Sagittal view, arrowhead showed the intimal flap of AA that was in the aorta root and arrow showed the artificial aortic valve. AA, ascending aorta; PA, pulmonary artery; DA, descending aorta.

A 53-year-old man presented with acute chest pain and shortness of breath for 12 h was admitted to our hospital. He had a history of hypertension and diabetes mellitus and underwent mechanical aortic valve replacement for hypertension associated chronic severe aortic valve regurgitation 18 months ago. The blood tests on admission are shown in Table 1 and most laboratory parameters deviated from the normal range. The bedside echocardiography confirmed the normal function of the mechanical aortic valve with an ejection fraction of 50%. The patient was examined by CT angiography (CTA) after he was stabilized by medical treatment and it showed a simultaneous ascending aortic dissection extending to descending aorta and PAD combined by aortopulmonary fistula (Figure 1).

**Table 1:** Primary blood tests of the patient on admission.

**Variables**	**Value**	**Normal range**
Leukocytes (×10^9^/L)	10.59	3.50–9.50
Neutrophils (×10^9^/L)	9.42	1.80–6.30
Hemoglobin (g/L)	129	130–175
Platelets (×10^9^/L)	91	125–350
Prothrombin time (s)	19.7	11.5–14.5
D-dimer (μg/mL)	6.83	<0.50
Alanine aminotransferase (U/L)	45.0	4.0–41.0
Estimated glomerular filtration rate (ml/min/1.73 m^2^)	32.7	>90.0
Creatinine (μmol/L)	188.0	59.0–104.0
Potassium (mmol /L)	5.23	3.50–5.10
Procalcitonin (ng/mL)	0.78	<0.50
Brain natriuretic peptide (pg/mL)	3,515	5–121
Cardiac troponin T (ng/mL)	1.88	<0.028

After we informed the families of the patient regarding the different treatments and related risks, including coronary artery involvement in aortic root dissection, renal malperfusion, heart failure, and especially complications associated with secondary cardiac surgery, the families and patient demanded surgical treatment.

Eventually, surgery was performed with median sternotomy and cardiopulmonary bypass was established *via* the intubation of the right atrial and right axillary artery, which was also used for selective cerebral perfusion in aorta arch replacement. The aorta root replacement and proximal anastomosis were performed first combined by removing the coronary arteries and reattaching them on the Dacron graft; in the meantime, the aortopulmonary fistula was closed with continuous 5-0 monofilament suture. Then, the hemiarch replacement and open distal anastomosis were completed under deep hypothermic circulatory arrest (nasopharyngeal temperature: 24°C). The surgery time was 474 min, cardiopulmonary bypass time was 185 min, cross-clamp time was 79 min, and deep hypothermic circulatory time was 23 min. The patient was transferred to the intensive care unit for further treatment and monitoring postoperatively. In the early period, there were no signs of unstable hemodynamics, respiratory insufficiency, and neurological disorders, and the ventilator weaning was performed 2 days after surgery. However, the patient was reintubated 10 days post-operatively due to dyspnea and methicillin-resistant Staphylococcus aureus was detected in sputum culture. Aspergillus was also detected in sputum culture 17 days postoperatively. Although vancomycin and fluconazole were used, the patient died of infectious shock 1 month after surgery.

## Discussion

According to the study by Jelani and Nosib (6), ascending aortic dissection could rarely lead to aortopulmonary fistula, which had been reported with 17 cases (13 acute and 4 chronic) until 2021 and further progression to PAD was an exceedingly rare condition. A case from Lempel et al. proposed a hypothesis for the etiology of the complex lesion (7). Ascending aorta and pulmonary artery trunk had a common adventitia, then extravasation of blood from aorta would flow to the adventitial space of pulmonary artery and cause compression, and finally invaded the pulmonary artery (7). Although it is difficult to confirm this process currently, there is often a channel between the aorta and pulmonary artery in previous cases of TAAD and PAD (3–5), which suggests that the hemodynamic factor of the formation of PAD came from the aorta in the complex entities.

It was reported that the known aortic valve disease and recent aortic manipulation were risk factors of AAD (8), which indicated the damage and pathological remodeling in the aortic wall. Khatchatourian and Vala also reported that a patient with TAAD was found PAD in the routine postoperative CTA after emergency surgery (9). Therefore, the history of aortic valve replacement, in this case, might be related to this series of entities and reminded us of careful examination of the area around the aorta regardless of the level of image or surgery in the patient with TAAD with a history of aortic manipulation.

In the early years, most patients with TAAD combined with aortopulmonary fistula and/or PAD died and the outcomes improved in recent years (6). The total in-hospital mortality of the 13 acute cases was 38.5% (6). According to data from the International Registry of Acute Aortic Dissection (IRAD), surgical mortality of TAAD declined from 25 to 18% in the late 1990s to early 2010s (10). If PAD also existed, a preoperative acute left-to-right shunt and prolonged cardiopulmonary bypass time caused by the additional intraoperative process might affect the outcome. Besides, our case had a previous history of aortic valve replacement and cardiac reoperation undoubtedly posed a great challenge to the treatment. Nevertheless, surgery was the only definitive treatment for this entity currently and we undertook an emergency operation, while the function of the vital organs of the patients was deteriorating.

It was interesting that in a review with 150 cases with PAD, medical management alone achieved a success rate of almost 70%, while surgery alone achieved a success rate of almost 90%. Meantime in a case series, three patients developed PAD induced by pulmonary arterial hypertension and survived for nearly 10 years by medical management (11). Therefore, it suggests that the prognosis of the patients with TAAD and PAD mainly depends on the progress of management of TAAD.

In conclusion, TAAD with concomitant aortopulmonary fistula and PAD is an extremely rare and dangerous entity. This complex entity may be associated with the previous aortic manipulation. Emergency surgery is needed as the management of TAAD and the outcome of the patients also mainly depends on the treatment effect of TAAD currently.

## Data Availability Statement

The raw data supporting the conclusions of this article will be made available by the authors, without undue reservation.

## Ethics Statement

The studies involving human participants were reviewed and approved by Ethics Committee of the Tongji Hospital of Tongji Medical College of Huazhong University of Science and Technology, written informed consent was obtained from the patient's legal guardian/next of kin for the publication of any potentially identifiable images or data included in this article.

## Author Contributions

LS drafted the manuscript and responsible for the collection of data and analysis. SX and JL designed the study and revised the manuscript. All the authors read and approved the final manuscript.

## Conflict of Interest

The authors declare that the research was conducted in the absence of any commercial or financial relationships that could be construed as a potential conflict of interest.

## Publisher's Note

All claims expressed in this article are solely those of the authors and do not necessarily represent those of their affiliated organizations, or those of the publisher, the editors and the reviewers. Any product that may be evaluated in this article, or claim that may be made by its manufacturer, is not guaranteed or endorsed by the publisher.
